# In PC3 prostate cancer cells ephrin receptors crosstalk to β_1_-integrins to strengthen adhesion to collagen type I

**DOI:** 10.1038/srep08206

**Published:** 2015-02-03

**Authors:** Miao Yu, Jinghe Wang, Daniel J. Muller, Jonne Helenius

**Affiliations:** 1 Department of Biosystems Science and Engineering, ETH Zurich, 4058 Basel, Switzerland; 2 Center for Precision Engineering, Harbin Institute of Technology, Harbin 150001, China

## Abstract

Eph receptor (Eph) and ephrin signaling can play central roles in prostate cancer and other cancer types. Exposed to ephrin-A1 PC3 prostate cancer cells alter adhesion to extracellular matrix (ECM) proteins. However, whether PC3 cells increase or reduce adhesion, and by which mechanisms they change adhesion to the ECM remains to be characterized. Here, we assay how ephrin-A1 stimulates PC3 cells to adhere to ECM proteins using single-cell force spectroscopy. We find that PC3 cells binding to immobilized ephrin-A1 but not to solubilized ephrin-A1 specifically strengthen adhesion to collagen I. This Eph-ephrin-A1 signaling, which we suppose is based on mechanotransduction, stimulates β_1_-subunit containing integrin adhesion *via* the protein kinase Akt and the guanine nucleotide-exchange factor cytohesin. Inhibiting the small GTPases, Rap1 or Rac1, generally lowered adhesion of PC3 prostate cancer cells. Our finding suggests a mechanism by which PC3 prostate cancer cells exposed to ephrins crosstalk to β_1_-integrins and preferably metastasize in bone, a collagen I rich tissue.

Eph receptors (Ephs) and ephrins constitute an important class of cell signaling proteins that are involved in regulating a variety of biological processes including cell adhesion, migration, differentiation, and segmentation, the formation of tissue boundaries, the guidance of neuronal axons, and embryonic development^1^. Eph-ephrin binding induced signals also play important roles in long-term potentiation^2^,^3^, angiogenesis^4^, and cancer^5^. The critical roles of Ephs and ephrins in nerve regeneration and in tumor progression have led to strategies to therapeutically target them^6^. Ephs, which are the largest known subfamily of receptor protein-tyrosine kinases in vertebrates, are divided into A and B subclasses based on sequence homologies and ephrin binding preferences. The nine members of the Eph class A (EphA) and five members of the Eph receptor class B (EphB) bind preferentially but not exclusively to different ephrins^7^. Similarly to Ephs, ephrins are membrane proteins that are divided into A and B subclasses. Ephrin-A ligands are GPI-anchored and comprise six members while ephrin-B ligands are type I transmembrane proteins comprising three members^8^. Although affinities differ between subclasses of ephrin-As and EphAs, most ephrin-As activate most EphAs^9^,^10^.

Ephs on one cell bind ephrins on neighboring cells and induce bi-directional signals^11^. Such Eph-ephrin binding induced signaling, which may require a high local density of ephrins^12^, can regulate the adhesion of cells to the extracellular matrix (ECM) by modulating integrin activity^13^. Integrins, the main cell adhesion receptors for ECM proteins, are heterodimers composed of one integrin α- and one integrin β-subunit, both of which are type I transmembrane proteins. There are 18 integrin α- and 8 integrin β-subunits in mammalian cells, which are known to form 24 different integrins^14^. Different integrins have distinct, but often redundant, functions and frequently bind promiscuously to ECM proteins. Integrins are divided into four groups, of which three are based on binding specificities to ECM proteins (e.g. collagen, laminin and fibronectin). The fourth group of integrins is involved in leukocyte adhesion. Integrin-mediated cell adhesion is highly regulated and the receptors can switch between different affinity states for ligands^15^. Integrin activation, the shifting from lower- to higher-affinity states, is regulated by two key adaptor proteins, kindlin and talin, which bind to the cytoplasmic tail of integrin β-subunits^16^,^17^. Kindlin and talin together with other adaptor proteins, such as α-actinin, paxilin and vinculin, link integrins bound to the ECM to the actin cytoskeleton^18^,^19^. Cytoskeletal interactions also control the diffusion and clustering of integrins, and the formation of long-lived focal adhesions^20^, whereas cell surface expression of integrins is regulated *via* endocytosis. Integrin mediated adhesion is regulated by various signaling molecules including FAK, SRC, ILK and small GTPases^18^,^21^,^22^,^23^. Moreover, integrins are also known to regulate each other in a process referred to as integrin crosstalk^24^,^25^,^26^.

Atomic force microscopy (AFM)-based single-cell force spectroscopy (SCFS) enables the forces with which cells adhere to their environment to be quantified^27^,^28^,^29^. To measure cell adhesion force, single cells are bound to an AFM cantilever and used to measure the adhesion strength of the bound cell to tissue, another cell, or substrate (e.g. ECM protein, ligands; Supplementary Fig. 1). The system is sensitive enough to characterize both the contribution of individual cell adhesion molecules (CAMs) to the adhesion formation and adhesion strengthening of the entire cell. In conventional SCFS, the cell is non-specifically attached to the AFM cantilever to avoid activating cell surface receptors *via* ligand binding^27^,^28^. However, functionalization of the AFM cantilever with ligands allows specific surface receptors of a cell to bind and to be functionally activated^30^. With functionalized AFM cantilevers it becomes possible to quantify differences in the adhesion force of stimulated and non-stimulated cells^31^. This approach is used to quantify the extent to which the binding of particular cell surface receptors to the ligand-functionalized cantilever regulates the cell's adhesion to another substrate. Such crosstalk was demonstrated between collagen-binding α_1_β_1_-integrins and fibronectin-binding α_5_β_1_-integrins in HeLa cells using SCFS^31^.

It was reported that in response to soluble ephrin-A1 PC3 cells, a prostate cancer cell line, lower adhesion to fibronectin and round up^32^. *In vivo*, however, ephrin-A1 is anchored to the outer membrane of the cell where it can function as substrate for cancer cells. Therefore, we investigated the role of surface-attached and solubilized ephrin-A1 in determining cancer cell adhesion. Using SCFS, we find that PC3 cells exposed to soluble ephrin-A1 did not change their adhesion to ECM proteins. However, the binding of PC3 cells to surface bound ephrin-A1 markedly increased their adhesion strength to collagen I but not to fibronectin. We further observe that this PC3 cell adhesion to collagen I was mediated by β_1_-subunit integrins and increased in strength with time. Our finding that ephrin-A1 attached to a surface stimulates PC3 cells to crosstalk (signaling pathways) with integrins containing β_1_-subunits hints at a possible mechanism by which PC3 prostate cancer cells preferably metastasize in bone, whose major ECM protein is collagen I.

## Results

### PC3 cells adhere to ephrin-A1-coated surfaces

**Figure 1:**
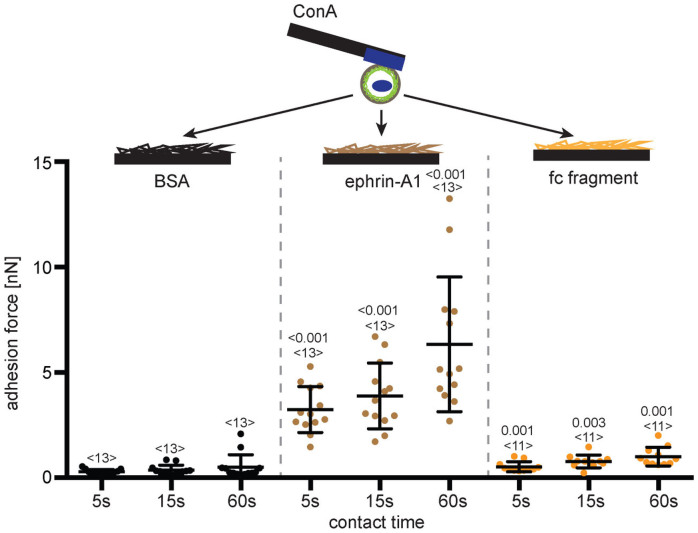
PC3 cells adhere to ephrin-A1-fc-coated surfaces. Top, depiction of the SCFS assay used to quantify the adhesion of PC3 cells. Single PC3 cells were bound to ConA-coated cantilevers and approached to BSA-, ephrin-A1-fc-, or fc-fragment-coated PDMS (secondary substrates). After a specified time the cantilever was retracted to detach the PC3 cell from the secondary substrate. During retraction the adhesion force of cell and secondary substrate was measured. Bottom, adhesion forces recorded for single PC3 cells during detachment from secondary substrates. Each dot represents the measurement of one cell with the number of cells assayed for each condition given by <n>. Indicated are the times (5, 15 and 60 s) the cell was in contact with the secondary substrate before being detached. Bars mark mean force and standard deviation. For each contact time, the statistical differences to control experiments (cell adhesion to BSA) were analyzed by Mann-Whitney *U*-tests (*P*-values given).

We wanted to characterize whether PC3 cells specifically adhere to ephrin-A1-coated surfaces. To quantify this adhesion we used AFM-based SCFS (Supplementary Fig. 1) and attached single suspended PC3 cells to the ends of concanavalin A (ConA) coated tip-less AFM cantilevers (primary substrate). The cantilever-bound cell was then pressed onto a fragment crystallizable (fc)-domain of immunoglobulin G (IgG), ephrin-A1-fc and BSA coated surface (secondary substrate) at a force of 2 nN and left to bind for a pre-determined contact time. Ephrin-A1-fc is the extracellular domain of ephrin-A1 fused to the fc domain of human IgG. The fc domain improved the immobilization of ephrin-A1 to the surface. At the end of the contact time, the cantilever was retracted to detach the cell from the secondary substrate. While retracting, the force acting on the cantilever was recorded. The maximum downward deflection of the cantilever recorded during the retraction of the cell measures the maximum adhesion strength and is referred to as the adhesion force. The adhesion forces of PC3 cells in contact with ephrin-A1-fc were considerably greater than of PC3 cells in contact with fc or BSA (Fig. 1). Moreover, the adhesion force of PC3 cells to ephrin-A1-fc increased by almost a factor of two when increasing the contact time from 5 to 60 s. These results showed that specific bonds between ephrin-A1 and presumably Ephs on the surface of PC3 cells are of sufficient strength to adhere cells to ephrin-A1 functionalized surfaces.

### Setting up the SCFS assay to characterize the crosstalk of ephrins to cell adhesion receptors

Having shown that PC3 cells bind to ephrin-A1 we asked whether the binding affects the adhesion of PC3 cells to ECM proteins. Thereto, we applied the previously developed stimulated SCFS assay (Supplementary Fig. 1), which is used to compare the adhesion of non-stimulated and stimulated cells to different substrates^31^. Particularly, we wanted to quantify the adhesion of PC3 cells attached to the cantilever by ConA and ephrin-A1-fc (primary substrates) to collagen I and fibronectin (secondary substrates). In order to increase the number of secondary substrates examined by SCFS, we used polydimethylsiloxan (PDMS) masks that allowed up to four different secondary substrate functionalizations in one Petri dish (Supplementary Fig. 1)^33^. Thus, a single PC3 cell, attached to a primary substrate coated cantilever, can be probed against several secondary substrates. For each secondary substrate, three SCFS measurements having contact times of 5, 15 and 60 s were always performed in this order with each cell. Because adhesion forces vary more between cells than between adhesion cycles of one cell^34^, we performed the same measurements with each primary substrate using at least three cells on a given day. The same measurements were performed on at least three separate days. Using this setup, the adhesion of PC3 cells in different experimental conditions was systematically examined.

### Ephrin-A1 binding stimulates PC3 cells to strengthen adhesion to collagen I

**Figure 2:**
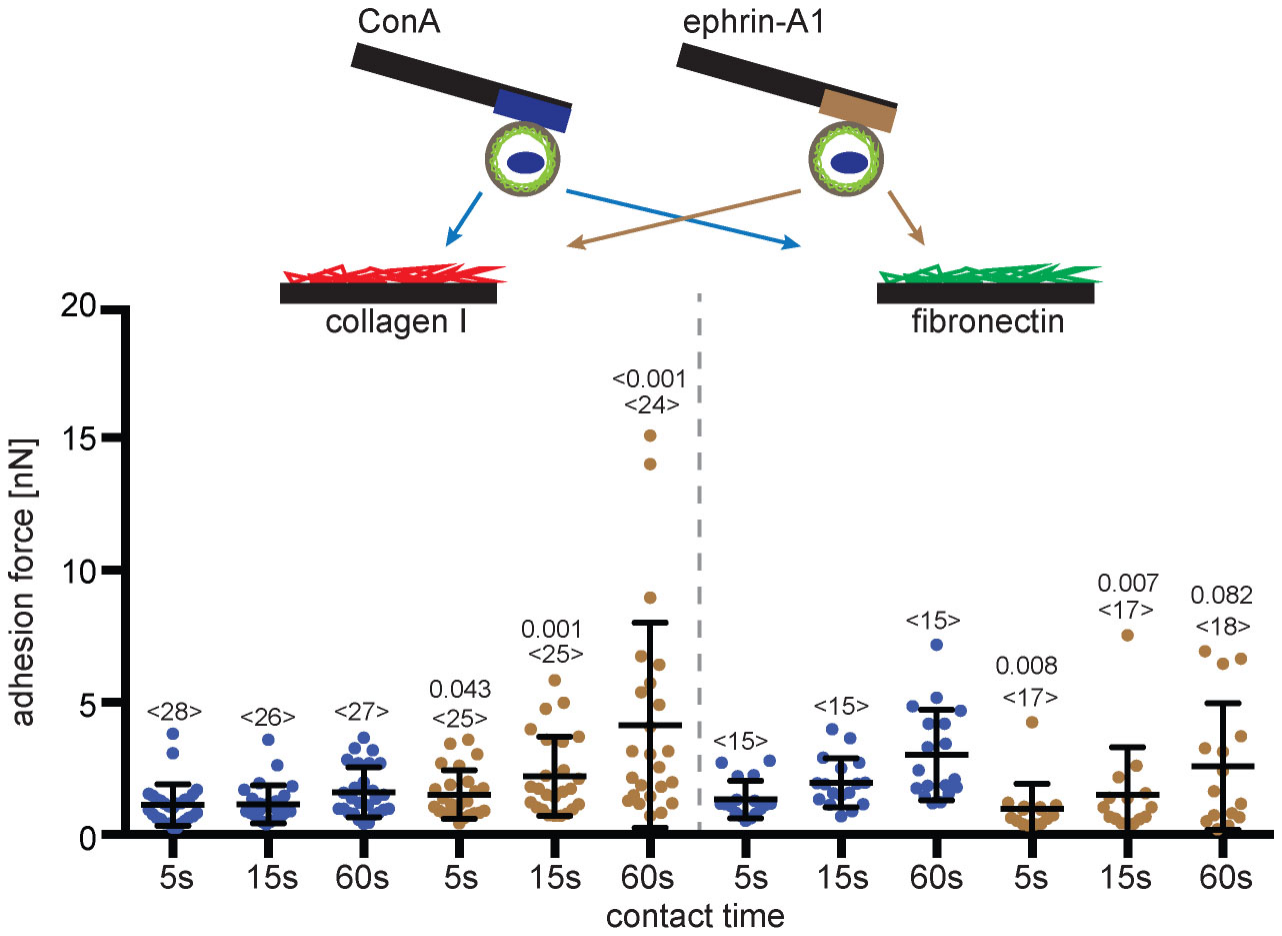
Ephrin-A1 stimulates PC3 cells to strengthen adhesion to collagen I. Top, depiction of SCFS experiments characterizing the ephrin-A1 induced crosstalk of PC3 cells. PC3 cells were bound to AFM cantilevers using either ConA (blue) or ephrin-A1-fc (brown) as primary substrates. Cells were approached to secondary substrates, which were collagen I and fibronectin coated PDMS surfaces in Petri dishes. Bottom, adhesion forces recorded for single PC3 cells during their detachment from secondary substrates, collagen I and fibronectin. Times (5, 15 and 60 s) give the contact time of cell and secondary substrate before being detached. Each dot represents the measurement of one PC3 cell attached to ConA (blue) or ephrin-A1-fc (brown) coated cantilevers. The number of cells assayed for each condition is given by <n>. Bars mark mean force and standard deviation. For each contact time, the statistical differences to control experiments (ConA bound cells) were analyzed by Mann-Whitney *U*-tests (*P*-values given).

Given the importance of ephrins and Ephs to cell adhesion^35^, we used the SCFS adhesion assay to study the effect of ephrin-A1 binding on the affinity of PC3 cells for collagen I and fibronectin (Fig. 2). For these adhesion experiments, suspended PC3 cells were added to Petri dishes functionalized with the secondary substrates; collagen I, the main component of the organic part of bone^36^, and fibronectin, the most common ECM component. Single PC3 cells were attached to cantilevers functionalized with either ConA or ephrin-A1-fc (primary substrates). After attachment, cells were left for at least 10 minutes to allow them to establish strong adhesion to the cantilever. Then SCFS adhesion assay cycles were performed as described above for all combinations of primary and secondary substrates and the adhesion forces determined. PC3 cells bound to ephrin-A1-fc adhered considerably stronger (factor ≈2 at 60 s contact time) to collagen I than PC3 cells bound to ConA, while the adhesion of cells to fibronectin did not depend on the primary substrate (Fig. 2). This ephrin-A1-fc dependent strengthening of the cell adhesion became dominant with increasing contact time to collagen I. At contact times longer than 60 s the PC3 cell often adhered stronger to collagen I than to the ephrin-A1-fc coated cantilever causing the cell to detach from the cantilever (Supplementary Fig. 2). To avoid detaching the cell from the cantilever we limited the contact time to 60 s or less.

We bound cells to cantilevers using ConA because this appears not to induce outside-in signaling in the attached cell^37^. Nevertheless, we asked whether the adhesion of PC3 cells could be affected by their attachment *via* ConA to the AFM cantilever. To test if ConA binding specifically affects the adhesions of PC3 cells we compared ConA with Cell-Tak bound cells and found no difference in the cells adhesion to collagen I (Supplementary Fig. 3). Therefore, we consider ConA bound cells to be non-stimulated and that any difference observed when binding cells to cantilevers using other substrates are the result of substrate dependent signaling.

In principle it is possible that the fc fragment of the ephrin-A1-fc construct contributes to the adhesion strengthening of PC3 cell to collagen I. To exclude this we conducted control SCFS experiments using the fc fragment of IgG as the primary substrate coating the AFM cantilever (Supplementary Fig. 3). The results showed that the fc fragment does not influence PC3 cell adhesion to collagen I.

Next, we asked whether PC3 cells increase adhesion force by increasing the contact area to the secondary substrate. To determine if the adhesion strengthening observed for ephrin-A1-fc bound PC3 cells was the result of increased contact area to collagen I, we imaged this contact area using confocal microscopy. Therefore, PC3 cells were incubated in Neuro-DiO, a green fluorescence membrane marker, and attached to an ephrin-A1-fc functionalized cantilever. Then, SCFS was performed while recording confocal images of the PC3 cell in contact with the collagen I coated surface (Supplementary Fig. 4). The cell-substrate contact area, as revealed from the fluorescence images, did not change during the contact phase of the adhesion measurement (Supplementary Fig. 4a,b). The same is true for PC3 cells, which have not been stimulated by ephrin-A1 (Supplementary Fig. 4c,d).

Taken together these experiments showed that PC3 cells sensing surface bound ephrin-A1 specifically strengthened adhesion to collagen I. That the strengthened adhesion was not the result of an increase in the substrate contact area leads to the conclusion that PC3 cells strengthen adhesion by regulating collagen I adhesion receptors at the secondary substrate contact. This implies that ephrin-A1 binding to PC3 cells initiates crosstalk to distinct CAMs.

### Only ephrin-A1 bound to a surface stimulates PC3 cells to strengthen adhesion to collagen I

**Figure 3:**
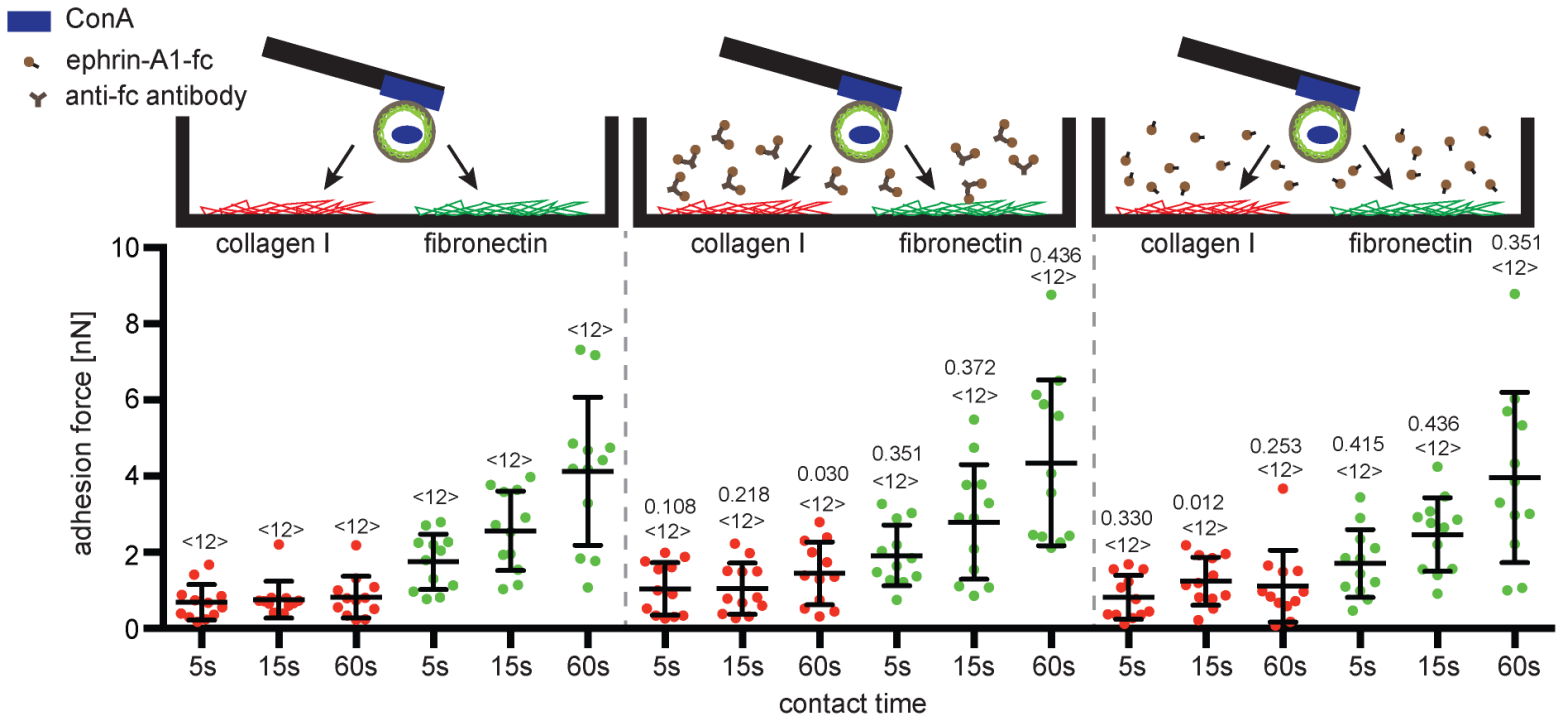
Immobilized ephrin-A1 is essential for strengthening the adhesion of PC3 cells to collagen I. Top, depiction of experimental conditions examined by SCFS. PC3 cells attached to cantilevers by ConA or ephrin-A1-fc. Cells were then approached to the secondary substrate collagen I (red) or fibronectin (green). In addition, PC3 cells were pre-incubated in media containing ephrin-A1-fc clustered by antibodies and solubilized ephrin-A1-fc before their adhesion to collagen I or fibronectin was measured. Bottom, adhesion forces recorded for single PC3 cells during their detachment from secondary substrates, collagen I and fibronectin. Times indicate the contact time of cell and secondary substrate before being detached. Each dot represents the measurement of one PC3 cell. The number of cells assayed for each condition is given by <n>. Bars mark mean force and standard deviation. For each contact time, the statistical differences to control experiments (measurements in absence of ephrin-A1-fc or antibody) were analyzed by Mann-Whitney *U*-tests (*P*-values given).

We observed that surface (cantilever) bound ephrin-A1-fc stimulated PC3 cell adhesion to collagen I whereas the adhesion to fibronectin remained unaffected. In apparent contrast to the latter finding, previous studies report that addition of ephrin-A1-fc to the cell media inhibits the adhesion of PC3 cells to fibronectin^32^. Also reported is that forms of ephrin-A1, soluble, clustered or surface bound, induce different cellular responses^38^. We conducted additional SCFS experiments to investigate whether the form of ephrin-A1-fc affects its ability to alter the adhesion of PC3 cells to collagen I or fibronectin. Thereto, PC3 cells were incubated with soluble ephrin-A1-fc (1 μg/mL) before their adhesion was measured. Alternatively, PC3 cells were incubated with ephrin-A1-fc that had been clustered by pre-incubation with anti-fc antibody (1:1) for 30 minutes on ice. In our assay, incubating PC3 cells with soluble and clustered ephrin-A1-fc did neither enhance cell adhesion to collagen I nor to fibronectin (Fig. 3). The slight decrease in PC3 cell adhesion to collagen I in the presence of solubilized ephrin-A1 is not statistically significant. Importantly, these experiments demonstrate that ephrin-A1 must be immobilized to stimulate the adhesion of PC3 cells to collagen I.

### Ephrin-A1 induced cell adhesion strengthening is specific to PC3 prostate cancer cells

**Figure 4:**
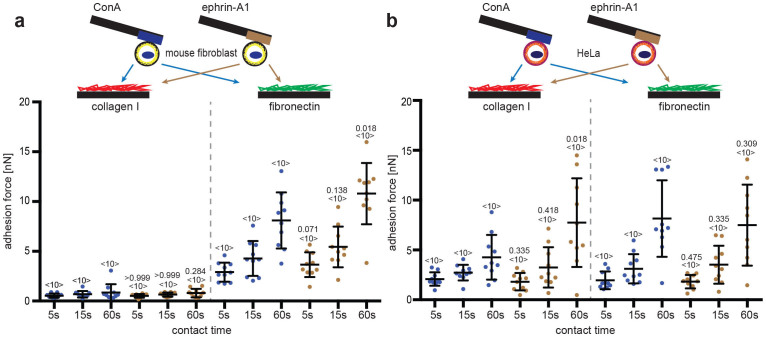
Ephrin-A1 fails to enhance adhesion of mouse fibroblasts and HeLa cells to collagen I. Top, depiction of SCFS experiments characterizing the ephrin-A1 induced crosstalk of (a) mouse fibroblasts and (b) HeLa cells. The cells were first bound to AFM cantilevers using either ConA or ephrin-A1-fc as primary substrates. Cells were then approached to secondary substrates, which were collagen I and fibronectin coated PDMS in Petri dishes. Bottom, adhesion forces recorded for single mouse fibroblasts and HeLa cells during their detachment from collagen I and fibronectin. Times give the contact time of cell and secondary substrate before being detached. Each dot represents the measurement of one cell attached to the cantilever *via* ConA (blue) or ephrin-A1-fc (brown). The number of cells assayed for each condition is given by <n>. Bars mark mean force and standard deviation. For each contact time, the statistical differences to control experiments (ConA bound cells) were analyzed by Mann-Whitney *U*-tests (*P*-values given).

Next, we asked whether the observed ephrin-A1 induced crosstalk to collagen I binding CAMs is common to mammalian cells or a distinct feature of PC3 cells. Therefore, we performed SCFS using mouse embryonic kidney fibroblasts and HeLa cells (Fig. 4). Both fibroblasts and HeLa cells adhered sufficiently well to primary substrates, ConA and ephrin-A1-fc, to perform SCFS. The adhesion forces to the different secondary substrates varied with cell line, with fibroblasts and HeLa cells binding fibronectin stronger than PC3 cells (Fig. 2 and 4). The adhesion of fibroblasts and HeLa cells to fibronectin increased with contact time. While mouse fibroblasts nearly failed to adhere to collagen I, HeLa cells adhered strongly. However, neither mouse fibroblasts nor HeLa cells showed statistically significant ephirn-A1 dependent adhesion changes. Next, we asked if ephrin-A1 induction was specific for prostate derived cells. Thereto, the ephrin-A1 induction was examined in three prostate cancer cell lines (LNCaP clone GFC derived form lymph node^39^, DU 145 from brain leson^40^ and MDA PCa 2b from bone metastasis^41^) and one prostate derived cell line (WPE1-NB26^42^) using our SCFS assay (Supplementary Fig. 5). Binding to immobilized ephrin-A1 failed to strengthen the adhesion to collagen I in all of these cell lines. Furthermore, none of the cell lines changed their adhesion to fibronectin (Supplementary Fig. 5). Taken together, these results indicate that ephrin-A1 dependent adhesion strengthening to collagen I is distinct to PC3 cells.

### Binding of ephrin-A1 to PC3 cells does not affect adhesion to other ECM proteins

**Figure 5:**
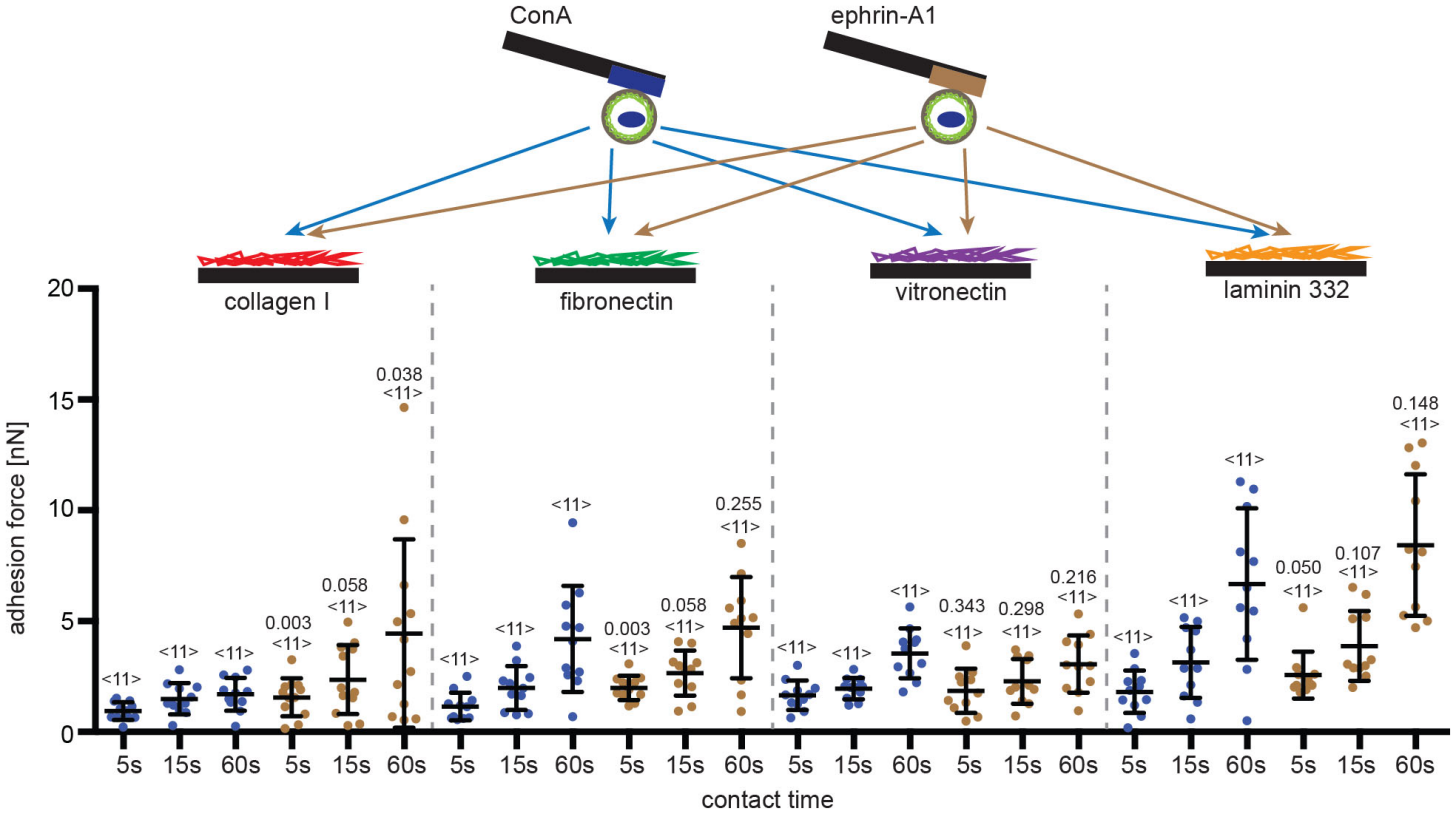
Ephrin-A1 induced adhesion strengthening of PC3 cells is specific for collagen I. Top, depiction of SCFS experiments examining the adhesion of PC3 cells to different secondary substrates, collagen I, fibronectin, vitronectin and laminin 332. PC3 cells were attached to the cantilever using either ConA (blue) or ephrin-A1 (brown) as primary substrates. Bottom, the adhesion forces recorded during the detachment of PC3 cells from secondary substrates. Indicated is the contact time of PC3 cell and secondary substrate. Each dot represents the measurement of one PC3 cell attached to the cantilever via ConA (blue) or ephrin-A1-fc (brown). The number of cells assayed for each condition is given by <n>. Bars mark mean force and standard deviation. For each contact time, the statistical differences to control experiments (ConA bound cells) were analyzed by Mann-Whitney *U*-tests (*P*-values given).

Next, we examined if ephrin-A1 binding influenced the adhesion of PC3 cells to ECM proteins other than collagen I. Thereto, fibronectin, vitronectin and laminin 332 were used as secondary substrates in the cell adhesion assay. Using SCFS, the strength with which single PC3 cells adhered to these ECM proteins and collagen I was quantified (Fig. 5). The cells adhered to each ECM protein with different strengths. However, the adhesion force to fibronectin, vitronectin and laminin 332 did not depend on whether the ephrin-A1-fc or ConA were used as primary substrates. This suggests that the ephrin-A1-induced enhancement of PC3 cell adhesion involves collagen I specific adhesion receptors.

### Adhesion of PC3 cells to collagen I is strengthened by integrins containing β_1_-subunits

**Figure 6:**
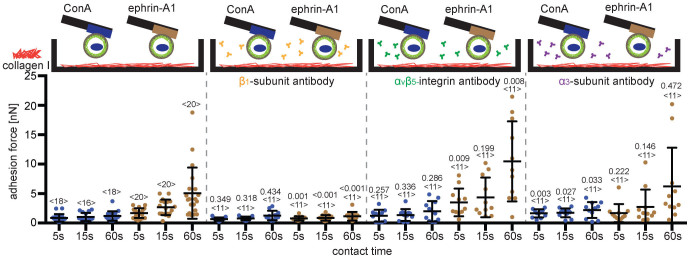
Ephrin-A1-activated PC3 cells strengthen adhesion to collagen I *via* integrin β_1_-subunits. Top, depiction of SCFS experiments quantifying the effect of adding antibodies (10 μg/mL) against integrin β_1_-subunit, α_v_β_5_-integrin and integrin α_3_-subunit on the adhesion of PC3 cells to collagen I. PC3 were attached to the cantilever using either ConA (blue) or ephrin-A1 (brown) as primary substrate. Bottom, adhesion forces recorded for ConA and ephrin-A1 bound PC3 cells during their detachment from collagen I secondary substrates. Indicated is the time that the cell was in contact with collagen I. Each dot represents the measurement of one PC3 cell attached to the cantilever via ConA (blue) or ephrin-A1-fc (brown). The number of cells assayed for each condition is given by <n>. Bars mark mean force and standard deviation. For each contact time, the statistical differences to control experiments (measurements made in the absence of antibody) were analyzed by Mann-Whitney U-tests (*P*-values given).

α_1_β_1_-, α_2_β_1_-, α_10_β_1_- and α_11_β_1_-integrins bind collagen I^43^. To determine if these integrins mediate adhesion of PC3 cells to collagen I and are responsible for the ephrin-A1-induced strengthening of cell adhesion we used integrin-blocking antibodies. Suspended PC3 cells were incubated in media containing antibodies against integrin β_1_-subunits^44^, integrin α_3_-subunits or α_v_β_5_-integrin, before their adhesion to collagen I was assayed (Fig. 6). Again, ConA and ephrin-A1-fc were used as primary substrates. As expected, the integrin β_1_-subunit antibody blocked the ephrin-A1-induced strengthening of cell adhesion to collagen I while the other antibodies did not. This blocking was efficient as the adhesion of PC3 cells to collagen I failed to substantially increase with contact time. Surprisingly, we found an increase in collagen I binding of ephrin-A1-bound PC3 cells treated with α_v_β_5_-integrin antibodies. This increase was not observed in ConA bound cells. In summary, the suppression of PC3 cell adhesion to collagen I by integrin β_1_-subunit antibodies indicated that ephrin-A1-binding to PC3 cells stimulated adhesion mediated by integrins containing β_1_-subunits.

### Targeting signaling proteins involved in the ephrin-A1 induced crosstalk

**Figure 7:**
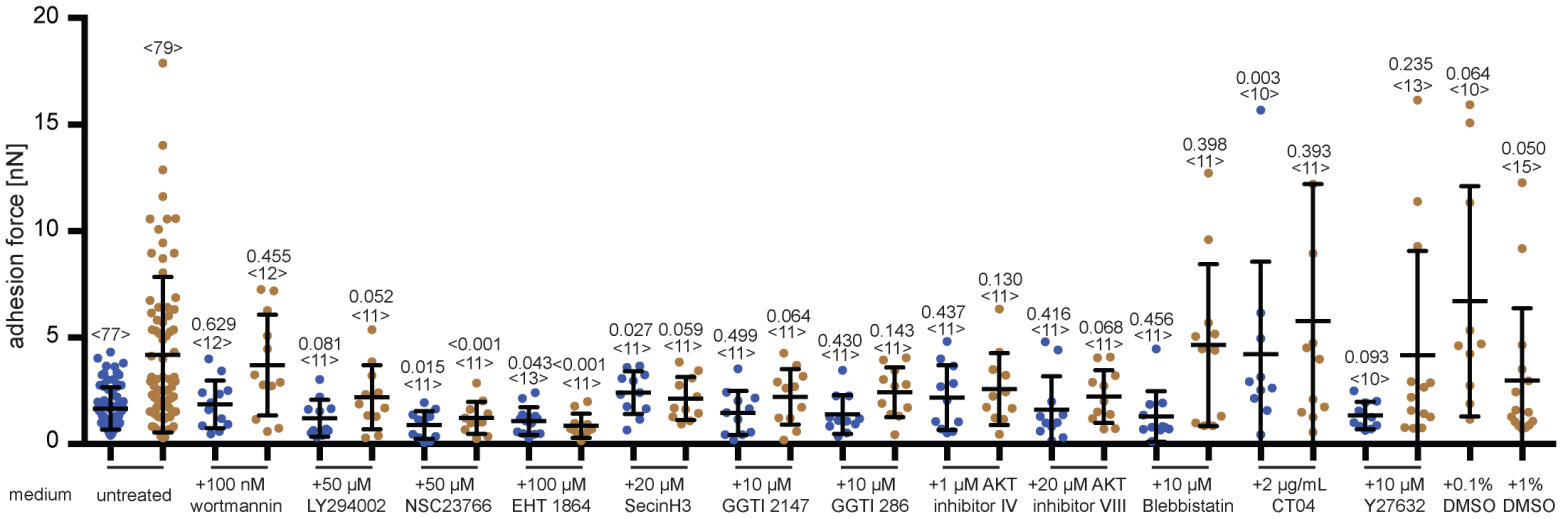
Ephrin-A1 activated strengthening of cell adhesion involves several signaling pathways. Graph depicts the adhesion force of PC3 cells after being in contact with collagen I for 60 s in the presence of inhibitors; wortmannin (100 nM, 0.01% final DMSO concentration), LY294002 (50 μM, 0.5%), NSC23766 (50 μM, 0.1%) and EHT1864 (100 μM, 0.13%), SecinH3 (20 μM, 0.1%), Akt inhibitor IV (1 μM, 0.1%), Akt inhibitor VIII (20 μM, 0.1%), GGTI2147 (10 μM, 1%), GGTI286 (10 μM, 1%), blebbistatin (10 μM, 0.02%), CTO4 (2 μg/mL, 1% glycerol), and Y27632 (10 μM, 0.1%). Blue and brown data points represent cells bound to ConA and ephrin-A1 coated cantilevers, respectively. Adhesion values recorded at contact times of 5 and 15 s are given in Supplementary Fig. 6. Each dot represents the measurement of one PC3 cell. The number of cells assayed for each condition is given by <n>. Bars mark mean force and standard deviation. Statistical differences to control experiments (untreated cells) were analyzed by Mann-Whitney *U*-tests (*P*-values are given). Perturbations were considered significant if all inhibitors targeting the same protein significantly reduced the adhesion force.

To examine the mechanism by which ephrin-A1 binding enhances the adhesion of PC3 cells to collagen I, we inhibited proteins that are possibly involved in the signal transduction of this crosstalk^6^,^35^. We inhibited PI3K, which in some signaling cascades is down stream of EphA2 and an effector of integrins^7^,^45^,^46^, with wortmannin and LY294002^47^,^48^, Rac1 with NSC23766 and EHT 1864^49^,^50^, Rap1 with GGTI2147 and GGTI286^51^, Akt with Akt inhibitor VIII and IV^52^,^53^, cytohesins with SecinH3^54^, myosin II with blebbistatin^55^, RhoA with CT04^56^, and ROCK with Y27632^57^. All inhibitors were used at sub-lethal concentrations (Supplementary Fig. 6). CT04 was added to spread PC3 cells 4 hours prior to performing adhesion assays, while all other inhibitors were added to suspended PC3 cells 30 minutes prior to performing adhesion assays. The inhibitors of Rac1, Rap1, cytohesin, and Akt all reduced the ephrin-A1-induced strengthening of PC3 cell adhesion to collagen I, while inhibitors of PI3K, myosin II, RhoA and ROCK did not (Fig. 7 and Supplementary Fig. 7). However, the inhibitors of Rac1 and Rap1 also lowered the baseline adhesion of both ephrin-A1 and ConA bound cells to collagen I. This indicates that these Rac1 and Rap1 are likely not specific for the ephrin-A1 stimulated signaling pathway but necessary for PC3 cell adhesion to collagen I. While the inhibitor results provide only limited insights into the ephrin-A1 induced signaling pathway, the finding that inhibiting either ROCK or RhoA has no effect suggests that the ROCK/RhoA pathway is not involved in establishing and strengthening PC3 cell adhesion to collagen I.

## Discussion

We applied SCFS to address how ephrin-A1 changes the adhesion of PC3 cells to ECM proteins. Thereto, single cells were bound to an ephrin-A1-coated AFM cantilever and their adhesion to different ECM proteins was probed. We found that PC3 cells bound to immobilized ephrin-A1 adhered stronger to collagen I than control cells, whereas adhesion to fibronectin was not altered. The finding that PC3 cell adhesion to fibronectin remained unaffected in the presence of either surface bound or soluble ephrin-A1, is in apparent contrast to a previous study reporting that addition of ephrin-A1-fc to the media inhibits PC3 cell adhesion to fibronectin^32^. Because the latter study did not directly measure cell adhesion, but inferred changes in cell adhesion from ephrin-A1 induced changes of PC3 cell morphology, we assume that the morphological changes are based on effects other than changes in adhesion.

### Ephrin-A1 induces PC3 cells to increase their β_1_-subunits integrin dependent adhesion to collagen I

Ephrin-A1 induced adhesion strengthening of PC3 cells to collagen I was abolished by integrin β_1_-subunit blocking antibodies. Therefrom, we conclude that in response to ephrin-A1, PC3 cells increased their adhesion to collagen I *via* β_1_-subunit containing integrins. The role of the integrin β_1_-subunit was predicted because collagen I is bound by α_1_β_1_-, α_2_β_1_-, α_10_β_1_- and α_11_β_1_-integrins^43^. The antibody mediated blocking of the β_1_-subunit may have induced compensation *via* activation of other integrins, which may have altered the adhesion of the cells to other integrin substrates^58^,^59^. However, because of our emphasis on collagen I adhesion this subject was not addressed.

### Ephrin-A1 stimulated PC3 cells increase the avidity of β_1_-subunits integrins

After 60 s of contact to collagen I about one third of the ephrin-A1 stimulated PC3 cells established high adhesion forces while the rest showed adhesion forces comparable to non-stimulated control cells (Fig. 2, 5, 6 and 7). Such large variations in early cell adhesion have been attributed to cells switching to an enhanced adhesion state^60^,^61^. Immuno-fluorescence microscopy of β_1_-integrins suggests that ephrin-A1 binding increased the cell surface expression of β_1_-integrins (Supplementary Fig. 8). These results indicate that Eph signaling, initiated by ephrin-A1 binding, induces PC3 prostate cancer cells to transition to an enhanced adhesion state by increasing the avidity of β_1_-integrins.

### Stimulation of PC3 cell adhesion depends on the mechanical state of ephrin-A1

Other studies of PC3 cells show ephrin-A1 stimulation having a different effect on cell adhesion. The addition of soluble ephrin-A1 induces cell rounding, inhibits cell migration, promotes the formation of retraction fibers, and suppresses integrin function^32^,^62^. This argues that stimulation by solubilized ephrin-A1 negatively regulates cell adhesion to the ECM. However, these studies lacked quantitative adhesion measurements and, hence, different cellular processes, such as cell contraction may cause the observed cell rounding and reduction of cell adhesion. Importantly, our SCFS experiments show that the adhesion of PC3 cells is stimulated differently depending on the ephrin-A1 form. Only surface bound ephrin-A1 stimulated PC3 cells to strengthen their adhesion to collagen I. Such a scenario mimics *in vivo where* ephrin-A1 is bound to cell surfaces. In contrast, soluble and antibody clustered ephrin-A1 did not enhance PC3 cell adhesion to collagen I. This is interesting as Eph signaling is known to depend on the form of ephrin-A1^12^,^63^. Furthermore, that only surface bound ephrin-A1 stimulates PC3 cell adhesion (Fig. 3), suggests that Eph-ephrin-A1 signaling depends on mechanotransduction.

Our SCFS setup did not allow quantitative adhesion measurements at contact times longer than 60 s, because after such contact times, the cells adhered too strongly to collagen I and detached from the cantilever. Therefore, we could only ascertain the initial adhesion of PC3 cells to ECM proteins and not examine the overall effect of Eph-ephrin signaling on mature adhesion. Although immobilized ephrin-A1 considerably stimulated PC3 cells to strengthen adhesion within the first 60 s of contact to collagen I, the long-term influence of ephrin-A1 stimulation on adhesion is likely more complex.

### Signaling molecules participating in the crosstalk between Eph and integrin

PC3 cells express mainly EphA2, for which ephrin-A1 is a ligand^32^. Thus, we assume that the enhanced PC3 cell adhesion is due to signaling by EphA2. To elucidate the signaling cascade(s) involved in ephrin–A1 induced adhesion strengthening we perturbed different signaling molecules. Although we were unable to dissect the signaling pathway, our results reveal several of its aspects. Since the inhibition of ROCK and RhoA had no effect on signal transduction, they are likely not involved in the early effects of ephrin-A1 induced cell adhesion strengthening. As myosin II can be regulated by a ROCK dependent pathway^64^, the inability of the myosin II inhibitor blebbistatin to affect the PC3 cell adhesion strengthening (Fig. 7) further substantiates the independence on RhoA. In apparent contrast, earlier studies indicate that RhoA is required for the ephrin-A1 dependent regulation of long-term cell adhesion^65^. However, as our experiments are limited to early (≤60 s) PC3 cell adhesion events, we cannot rule out that Rho-family GTPases, which are central to integrin mediated adhesion signaling and crosstalk^22^, play a dominant role at longer adhesion times. Our experimental finding accord with the observation that during initial cell adhesion phases RhoA-GTP levels are reduced by the activation of Rac1, whereas during later adhesion phases the activity of Rac1 decreases and that of RhoA increases^22^. Accordingly, we observe that upon Rac1 inhibition, PC3 cell adhesion strengthening is abolished. This observation suggests that Rac1 is important for ephrin-A1 to switch PC3 cells to the strengthened adhesion state. However, since ConA bound non-stimulated PC3 cells were also less adhesive when Rac1 was inhibited (Fig. 7), Rac1 may be a general regulator of early cell adhesion and, therefore, not specific to ephrin-A1 induced adhesion.

In contrast to the Rac1 inhibitor, the cytohesin inhibitor SecinH3 did not weaken non-stimulated cell adhesion but abolished the ephrin-A1 induced strengthening of cell adhesion (Fig. 7). In HeLa cells, cytohesin 2 promotes recycling of β_1_-subunit containing integrins to the plasma membrane and cytohesin 3 down regulates cell adhesion^66^. Because cytohesin 1 and 4 are mainly expressed in immune cells^67^ and cytohesin 3 inhibits cell adhesion our results suggest that in prostate cancer cells cytohesin 2 activity is up-regulated upon ephrin-A1 stimulation. Our data also suggests that Rap1 is a part of the signaling induced by ephrin-A1 stimulation. Rap1, is a regulator of RIAM, which activate integrins by localizing talin to the plasma membrane^68^,^69^. However, as Rap1 inhibition reduces adhesion of non-stimulated and ephrin-A1 stimulated PC3 cells it may be a more general regulator of PC3 cell adhesion. Interestingly, we found that the early ephrin-A1 induced adhesion of PC3 cells was lowered by Akt inhibition, which contradicts a study showing that Akt2 and 3 activity reduce PC3 cell adhesion to collagen I^70^. Because Akt inhibition did not reduce the adhesion of non-stimulated PC3 cells we propose that Akt is only involved in enhancing ephrin-A1 stimulated adhesion to collagen I. These findings elucidate some possible molecules involved in the signaling pathway by which ephrin-A1 stimulates β_1_-integrin mediated adhesion to collagen I. We hope that more extensive cell biological studies will unravel in more details of how this ephrin-A1 dependent crosstalk regulates cell adhesion.

### Medical relevance of ephrin induced stimulation of prostate cancer cell adhesion

The finding that the prostate cancer cell line, PC3, responds to ephrin-A1 binding by strengthening adhesion to collagen I is of possible medical relevance. Prostate cancers metastasis to a very high percentage in bone^71^, the main protein component of which is collagen I. EphA2, which is over-expressed in PC3 cells^32^, regulates prostate cancer invasion and metastasis^72^. EphA2 is involved in cell invasion and metastasis of several cell lines and different cancer types *in vivo*^73^,^74^,^75^. In addition, *Taddei et al* show that EphA2 mutant cells do not present ephrin-A1 induced cell rounding, retraction fiber formation and *in vivo* metastasis^76^. Our results indicate, that EphA2 may not only have an important role in the delamination of cancer cells from the primary tumor but also in the process of metastasis formation. An implication of ephrin-A1 in prostate cancer progression is not evident, but ephrins such as ephrin-B2 are expressed in osteocytes and osteoblasts^77^. In osteoclast precursors and osteoblasts, the bidirectional signaling of EphA2 and ephrin-A2 regulates the initial phase of bone remodeling^78^. In this context, the enhanced early adhesion to collagen I upon EphA2 activation is possibly involved in the adhesion of prostate cancer cells to bone matrix.

## Methods

### Cell culture

The human prostatic carcinoma cell line, PC3, was maintained in 25 mM HEPES RPMI 1640 medium (Sigma-Aldrich) supplemented with 10% (v/v) fetal bovine serum (FBS, Sigma-Aldrich), 1 mM sodium pyruvate, 100 units/mL penicillin and 100 μg/mL streptomycin (Gibco-Life Technologies). HeLa-Kyoto cells were maintained in DMEM (Gibco-Life Technologies) supplemented with 10% FBS, 100 units/mL penicillin and 100 μg/mL streptomycin. Mouse embryonic kidney fibroblasts were maintained in DMEM supplemented with 10% FBS, 100 units/mL penicillin and 100 μg/mL streptomycin.

### SCFS setup

For SCFS both a NanoWizard II AFM equipped with the CellHesion module and a CellHesion 200 (both JPK Instruments) mounted on inverted microscopes (Observer. Z, Zeiss) were used. During SCFS cells were maintained at 37°C using a Petri dish heater (JPK Instruments) or temperature controlled (Life Imaging Services) incubator box. 200 μm long tip-less V-shaped silicon nitride cantilevers having nominal spring constants of 0.06 N/m (NP-0, Bruker) were used. Cantilever spring constants were determined using the equipartition theorem^79^.

### Surface coating of AFM cantilever and Petri dishes

Cantilevers were prepared as described previously^80^. In short, cantilevers were plasma-cleaned prior to an overnight incubation (at 4°C) in ConA (2 mg/mL, Sigma), fc-fragment (50 μg/mL) or ephrin-A1-fc (50 μg/mL) in PBS, or Cell-Tak (63 μg/mL, BD Biosciences). The cantilever bound substrate is referred to as primary substrate. Secondary substrates were prepared as follows: glass bottom Petri dishes (35 mm FluoroDish, World Precision Instruments) were coated with a polydimethylsiloxan (PDMS, Sylgard 184, Dow Corning) mask to allow four different substrates coatings^33^. Then, 16 μL collagen I (160 μg/mL, Inamed Biomaterials), bovine fibronectin (50 μg/mL, Merck), laminin 332 (laminin 5, 50 μg/mL, Abcam) or vitronectin (from human plasma, 50 μg/mL, Merck Millipore) in PBS was added to PDMS surfaces (area 9 mm^2^) for an overnight incubation at 4°C.

### Adhesion force measurements by SCFS

For adhesion force measurements, cells were grown to ≈80% confluency, washed with PBS, trypsinized with 0.25% trypsin-EDTA (Gibco-Life Technologies) for 3 minutes, pelleted, and suspended into CO_2_-independent serum-free RPMI 1640 (measurement medium). Cell suspensions were pipetted onto secondary substrate-coated supports and cells were allowed to settle. To attach single cells, the apex of a calibrated, primary substrate-functionalized cantilever was lowered with a velocity of 10 μm/s onto a cell until a contact force of 5 nN was detected. After 5 s of contact, the cantilever was raised from the Petri dish by 50 μm, where the cantilever-bound cell was incubated for >10 minutes^80^. For each adhesion measurement the cantilever-bound cell was lowered onto the secondary substrate (Supplementary Fig. 1a, I) until reaching a contact force of 2 nN, the cantilever height was maintained for a predetermined contact time of 5, 15 or 60 s (Supplementary Fig. 1a, II), and retracted from the secondary substrate by >90 μm (Supplementary Fig. 1a, III and IV). Cantilever approach and retract velocity was 5 μm/s. Each data set was generated using at least nine cells; thereto at least three cells for each condition assayed per day on at least three separate days. Only one adhesion measurement was performed for each cell for a given contact time secondary substrate combination. The order of contact times for each cell and secondary substrate combination was always: 5, 15 and 60 s. Cell recovery times between adhesion measurement cycles were never shorter than the contact time, even excluding the time necessary to raise and lower the cell. Detachment forces were extracted from force-distance curves (Supplementary Fig. 1b) recorded during each cell adhesion measurement using the JPK data processing software. For adhesion receptor crosstalk measurements cells were bound to cantilevers coated with different primary substrates (see cantilever preparation). Thereby, effects of the primary substrate on the adhesion properties of cells to a secondary substrate were addressed (Supplementary Fig. 1c).

### Antibody blocking and inhibitor assays

The antibodies, anti-human IgG (Fc) (BioConcept AG, Switzerland), AIIB2 (integrin β_1_-subunit blocking antibody), P5H9 (α_v_β_5_-integrin blocking antibody) and P1B5 (integrin α_3_-subunit blocking antibody; all 10 μg/mL supernatants; DSHB, Iowa) were incubated with cells on ice for 30 minutes prior to SCFS. To inhibit cell signaling, wortmannin (100 nM; Sigma-Aldrich)^47^, LY294002 (50 μM; Cell Signaling Technology)^48^, NSC23766 (50 μM; Merck Millipore)^49^, EHT 1864 (100 μM; R&D Systems)^50^, GGTI 2147 or GGTI286 (10 μM; Merck Millipore)^51^, Akt inhibitor IV (1 μM; Merck Millipore)^52^, Akt inhibitor VIII (20 μM; Merck Millipore)^53^, SecinH3 (20 μM; Merck Millipore)^54^, blebbistatin (10 μM; Merck Millipore)^55^ or Y-27632 (10 μM; Merck Millipore)^57^ were added to the measurement media at the concentrations given. Cells were then incubated for 30 minutes at 37°C prior to SCFS. All inhibitors were dissolved in DMSO and stored at −20°C.

### Inhibitor concentration assays

PC3 cells were grown in 96-well microtiter glass bottom plates (P96G-1.5-5-F, Mattek Corporation) in 10% FCS RPMI 1640 for 2 days before the media was exchanged with serum-free RPMI 1640. After 12 h inhibitors (see above) were added at different concentrations and DIC microscopy images of cells were recorded using an inverted microscope (Eclipse Ti, Nikon) Throughout, cells were maintained at 37°C and 5% CO_2_.

### Adhesion contact area

PC3 cells were detached with 0.25% trypsin-EDTA at 37°C for 3 minutes, spun down at 160 g for 3 minutes, suspended in 1 mL FCS-free RPMI 1640 containing 5 μl Neuro-DiO (CellBrite Green, Biotium) and incubated in 37°C for 20 minutes. The cells were washed twice in 1 mL of FCS-free RPMI 1640 media, by pelleting cells at 160 g for 5 minutes. SCFS was performed with Neuro-DiO labeled cells, while fluorescence images of the cell-collagen I contact area were recorded using confocal microscopy (LSM700, LCI 63×/1.3 objective, Zeiss).

### Statistical analysis

Statistical analysis was performed using the Prism4 software (GraphPad Software). All quantitative data (Fig. 1–6, and Supplementary Fig. 2, 3, 5, and 7) is shown with bars marking mean ± standard deviation. Significance was calculated using the Mann–Whitney *U*-test with *P* ≤ 0.05 being significant and *P* ≤ 0.01 very significant.

## Author Contributions

M.Y. conducted the experiments and analyzed the data together with J.H. J.W. supervised M.Y. J.H. and D.J.M. designed the experiments. M.Y., J.H., D.J.M. wrote the paper.

## Supplementary Material

Supplementary Information Supplementary Figures
