# Supplementary material for: In PC3 prostate cancer cells ephrin receptors crosstalk to β1-integrins to strengthen adhesion to collagen type I
Source: Sci Rep. 2015 Feb 3;5:8206. doi: 10.1038/srep08206 (PMC4314628; doi:10.1038/srep08206)
Supplement: Supplementary Information — Supplementary Figures [file srep08206-s1.pdf]

In PC3 prostate cancer cells ephrin receptors crosstalk to β_1-integrins to strengthen adhesion to collagen type I

Miao Yu[1,2], Jinghe Wang[2], Daniel J. Muller[1] & Jonne Helenius[1]

[1]Department of Biosystems Science and Engineering, ETH Zurich, Mattenstr. 26, 4058 Basel, Switzerland;

[2]Center for Precision Engineering, Harbin Institute of Technology, Harbin 150001, China

Supplementary Information

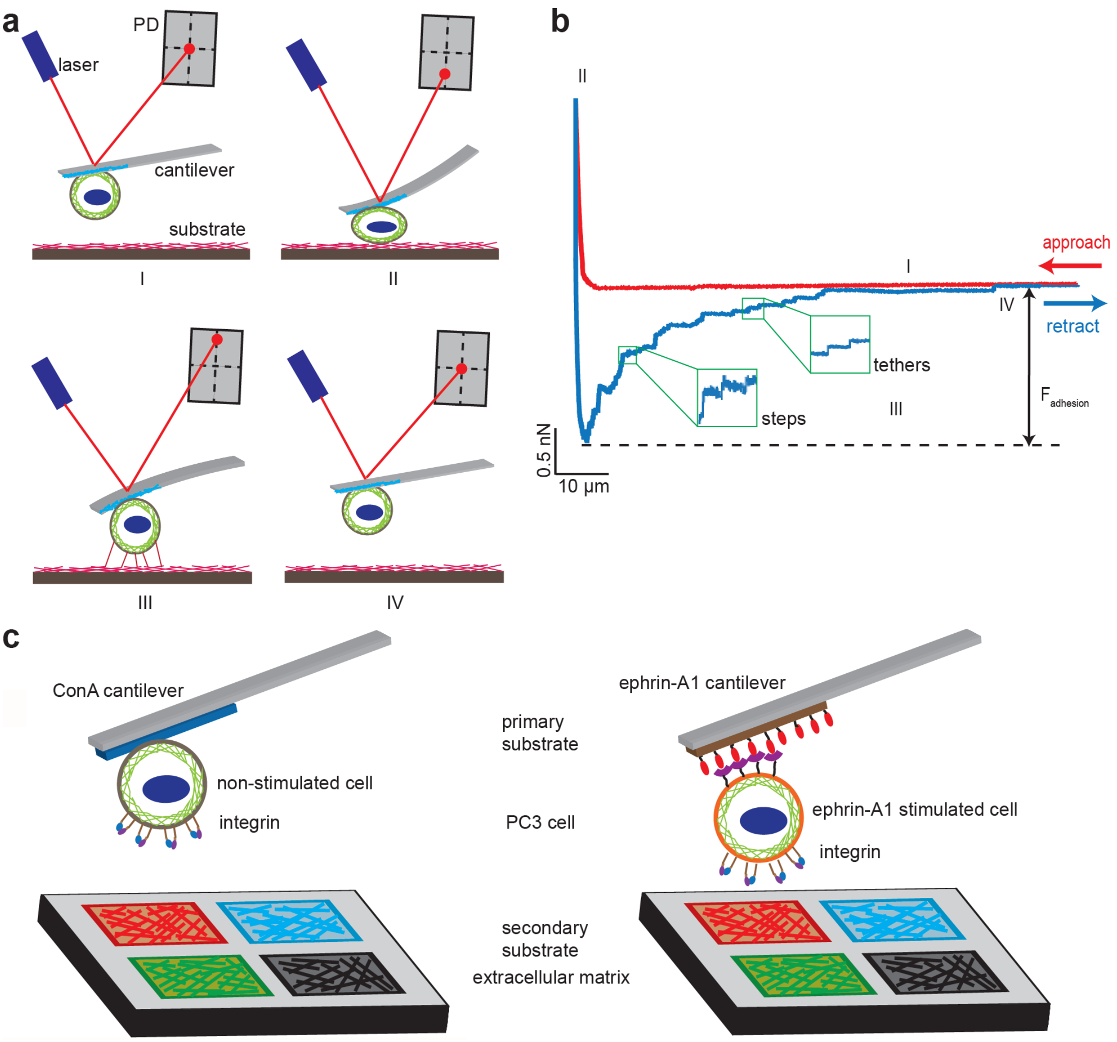

Supplementary Figure 1. SCFS setup. (**a**) A cell is attached to a coated micro-cantilever. To determine the force acting on the cell, the cantilever deflection is measured by reflecting a laser beam (red) off the cantilever onto a multi-segment photodiode (PD, gray). For adhesion force measurements, a cantilever-bound cell is approached to a substrate (I) until a preset force is reached (II). After a specified contact time, the cantilever is retracted (III) until cantilever-bound cell and substrate are completely separated (IV). During approach and retraction, force-distance curves are recorded. (**b**) Representative approach (red) and retraction (blue) force-distance curves. Typically force-distance curves show different features: In the approach curve (red) the cantilever deflection measured upon pressing the cell onto the substrate correlates with the stiffness of the cell. The retraction force curve (blue) records the adhesive forces of the cell, which represents the maximum downward force deflecting the cantilever and, thus, the maximum adhesion force ($F_{adhesion}$) needed to detach cell and substrate. After recording the maximum adhesion force, single receptor unbinding events are observed. Rupture events (here called steps) are recorded when the CAM-ligand bond of a cytoskeleton-linked CAM fails. Tether events are recorded when a membrane tether is extruded from the cell membrane with the CAM at its tip (tethers). In the latter case attachment of the CAM to the cytoskeleton is either too weak to resist the mechanical stress applied or non-existent. (**c**) SCFS setup for detecting ephrin-A1 stimulated PC3 cell adhesion. Cantilevers are coated either with ConA or ephrin-A1-fc as primary substrate. After attaching a PC3 cell to the cantilever, its adhesion to a secondary substrate is probed. Glass bottom petri dishes covered with a PDMS masks (gray) that allow four different secondary substrates to be coated.

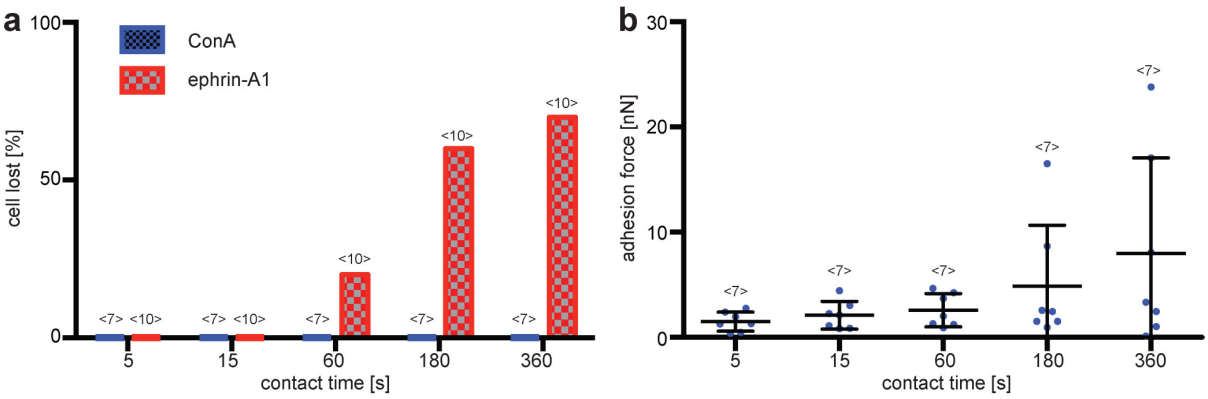

Supplementary Figure 2. Ephrin-A1 activated PC3 cells increase adhesion strength to collagen I with increasing contact time. (**a**) Percent of PC3 cells that detached from the cantilever (primary substrate) when being retracted from collagen I (secondary substrate). With increasing contact time to collagen I more PC3 cells detached from the ephrin-A1-fc coated cantilever (red bars). PC3 cells adhering to ConA coated cantilevers did not detach from collagen I (blue bars). (**b**) Adhesion force of single PC3 cells to collagen I when bound to the cantilever using ConA. Cell adhesion forces were examined using subsequent longer contact times of 5, 15, 60, 180 and 360 s. Each dot represents the adhesion measurement of one PC3 cell. The number of cells assayed for each condition is given by <n>.

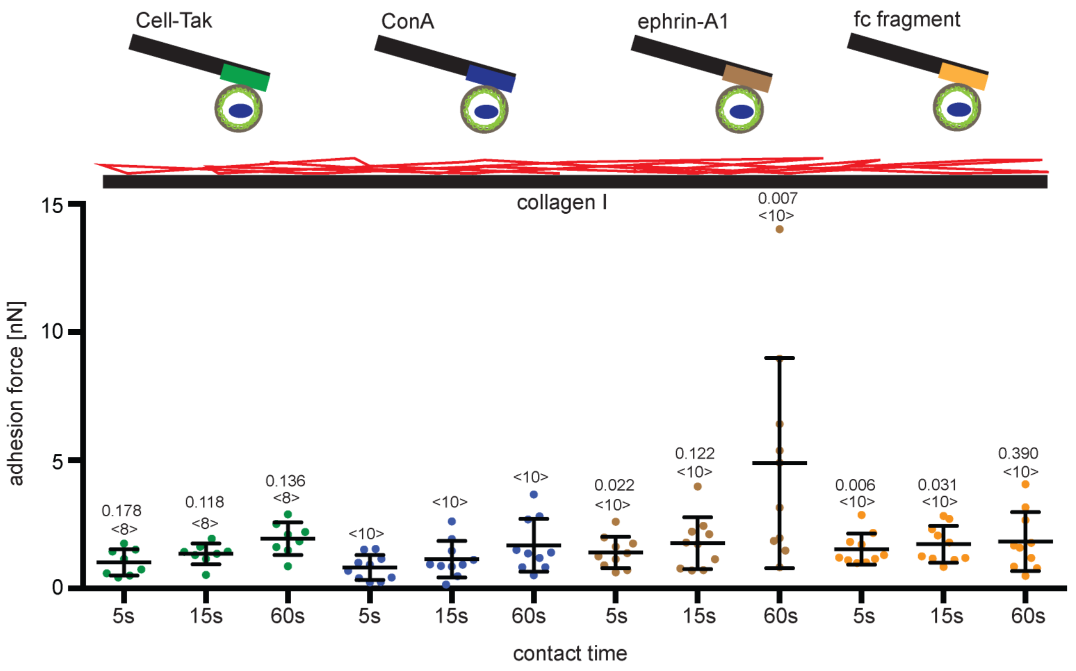

Supplementary Figure 3. PC3 cells attached to the AFM cantilever *via* Cell-Tak, ConA or fc fragment fail to strengthen adhesion to collagen I. Top, depiction of SCFS experiments characterizing the effect of different cantilever coatings on PC3 cell adhesion to collagenI. PC3 cells were bound to AFM cantilevers using either Cell-Tak (green), ConA (blue), ephrin-A1-fc (brown), or fc fragment (yellow). Cells bound to these primary substrates were approached to collagen I coated PDMS surfaces in Petri dishes. Bottom, adhesion forces recorded for single PC3 cells during their detachment from collagen I. Times (5, 15 and 60 s) indicate the time that the cell was in contact with collagen I before being detached. Each dot represents the measurement of one PC3 cell attached to Cell-Tak (green), ConA (blue), ephrin-A1-fc (brown), or fc (yellow) coated cantilevers. The number of cells assayed for each condition is given by <n>. Bars mark mean force and standard deviation. For each contact time, the statistical differences to control experiments (ConA bound cells) were analyzed by Mann-Whitney *U*-tests (*P*-values given in gray).

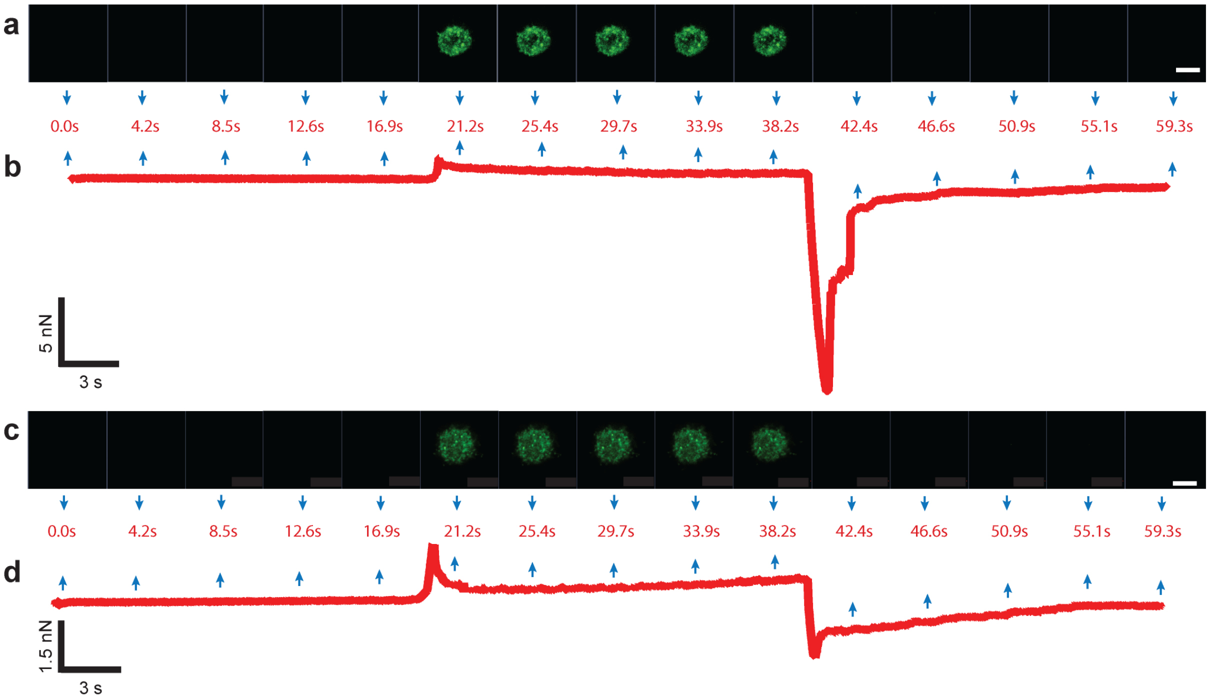

Supplementary Figure 4. Adhesion strengthening does not necessitate increase in cell-collagen I contact area. (**a**) Time-lapse confocal images of ephrin-A1 stimulated PC3 cells stained with Neuro-DiO. The focal plane was maintained at the height of the collagen I secondary substrate. At zero time the PC3 cell attached to the cantilever *via* ephrin-A1-fc was approached at 5 μm/s for ≈100 μm until it contacted collagen I coated PDMS with 2 nN contact force. After 20 s of contact the cell was retracted at 5 μm/s. (**b**) Force-time curve (red) recorded while recording images in (**a**). (**c**) Time-lapse confocal images of non-stimulated PC3 cells stained with Neuro-DiO. Experiments were conducted as described in (**a**) except the PC3 cell was attached to the cantilever using ConA. (**d**) Force-time curve recorded while recording images in (**c**). Scale bars (white), 10 μm.

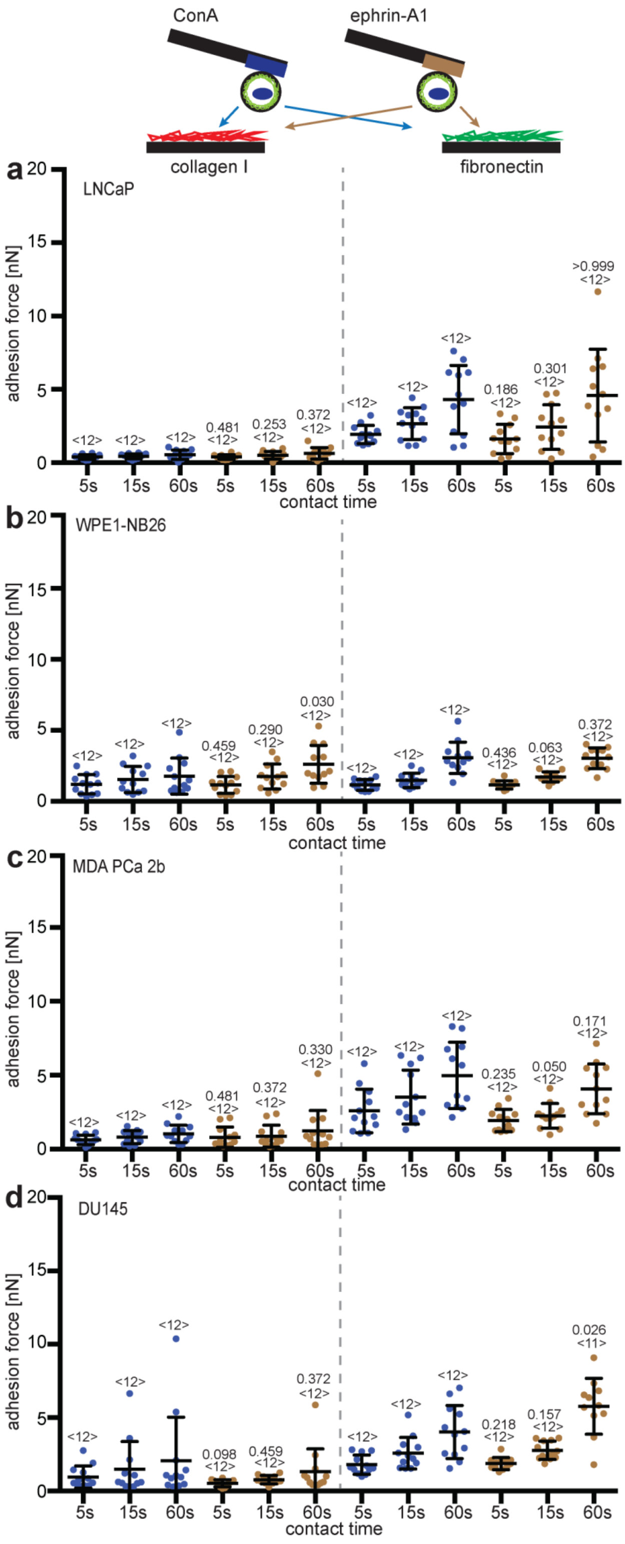

Supplementary Figure 5. Ephrin-A1 fails to enhance adhesion of other prostate cells to collagen I. Top, depiction of SCFS experiments characterizing ephrin-A1 induced crosstalk in (**a**) LNCaP (**b**), WPE1-NB26 (**c**), MDA PCa 2b and (**d**) DU 145 cells. The cells were first bound to AFM cantilevers using either ConA (blue data) or ephrin-A1-fc (brown data) as primary substrates. Cells were then approached to secondary substrates, which were collagen I and fibronectin coated PDMS in Petri dishes. Adhesion forces recorded for single LNCaP, WPE1-NB26, MDA PCa 2b and DU 145 cells during their detachment from collagen I and fibronectin are shown. Times denote the contact time of cell and secondary substrate before being detached. Each dot represents the measurement of one cell attached to the cantilever *via* ConA (blue) or ephrin-A1-fc (brown). The number of cells assayed for each condition is given by <n>. Bars mark mean force and standard deviation. For each contact time, the statistical differences to control experiments (ConA bound cells) were analyzed by Mann-Whitney *U*-tests (*P*-values given in gray).

LNCaP clone GFC cells (ATCC, US) were maintained in 25 mM HEPES RPMI 1640 medium (Gibco-Life Technologies) supplemented with 10% (v/v) fetal bovine serum (FBS, Sigma-Aldrich), 1 mM sodium pyruvate. WPE1-NB26 cells (ATCC, US) were maintained in Keratinocyte-SFM (Gibco-Life Technologies). DU 145 cells (ATCC, US) were maintained in EMEM (Sigma-Aldrich) supplemented with 10% FBS; MDA PCa 2b cells (ATCC, US) were maintained in BRFF-HPC1 (Enzo Life Sciences) supplemented with 20% FBS. All media also contained 100 units/mL penicillin and 100 µg/mL streptomycin (Gibco-Life Technologies).

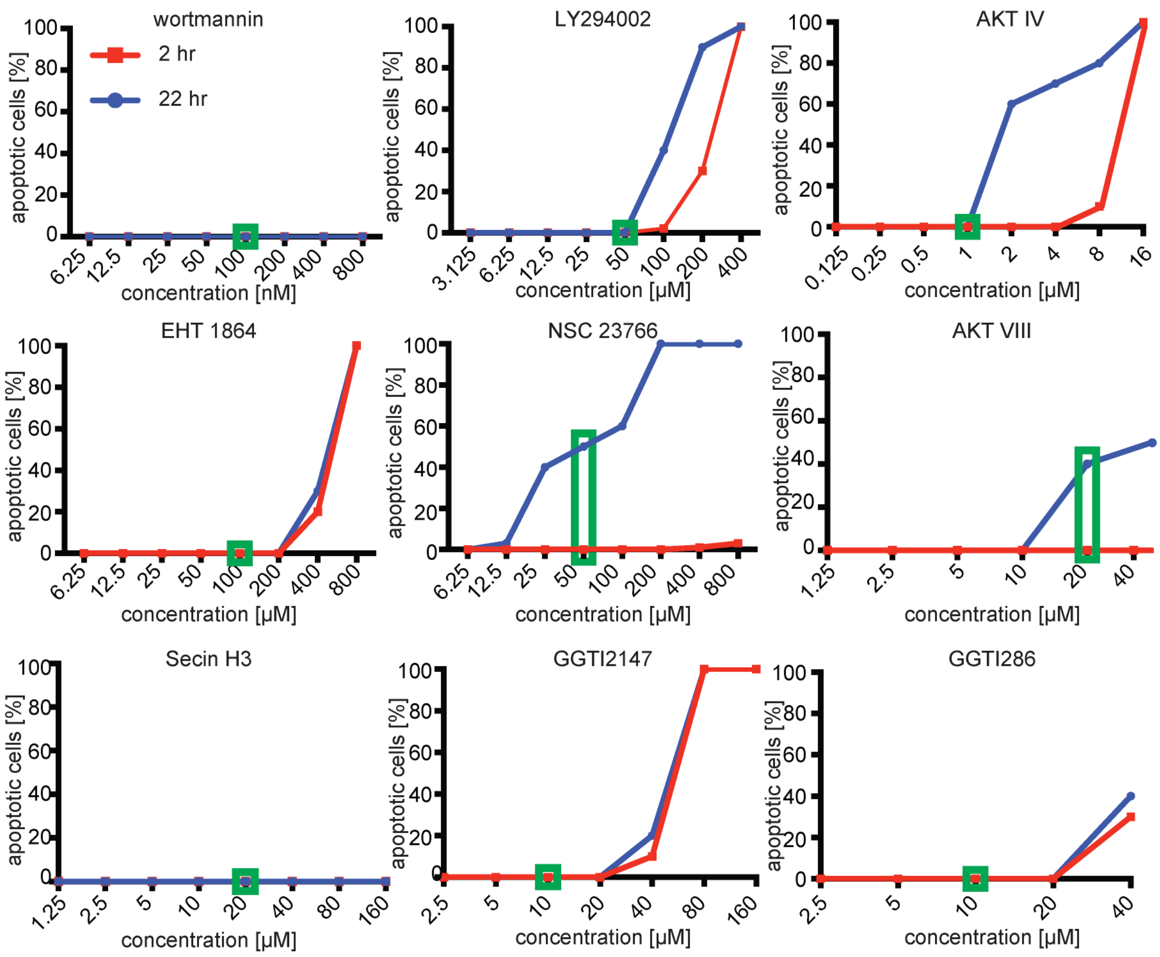

Supplementary Figure 6. The inhibitor concentrations used are not lethal. Shown are graphs of the viability of PC3 cells in serum-free RPMI 1640 media containing different concentrations of the inhibitors. Plotted is the percentage of deformed cells after 2 h (red) and 22 h (blue) of inhibitor treatment. The green box indicates the inhibitor concentration used for cell adhesion assays.

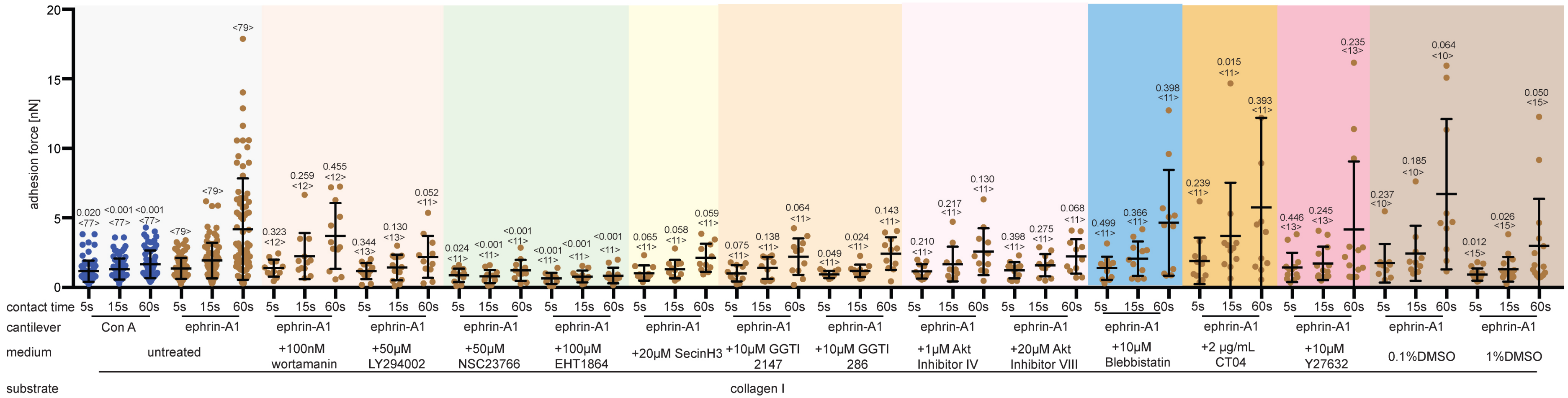

Supplementary Figure 7. Signaling pathways required for adhesion strengthening of PC3 cells to collagen I. Graph depicts the adhesion force of PC3 cells after 5, 15 and 60 s of contact with collagen I in the presence of inhibitors; wortmannin (100 nM, 0.01% final DMSO concentration), LY294002 (50 µM, 0.5%), NSC23766 (50 µM, 0.1%) and EHT1864 (100 µM, 0.13%), SecinH3 (20 µM, 0.1%), Akt inhibitor IV (1 µM, 0.1%), Akt inhibitor VIII (20 µM, 0.1%), GGTI2147 (10 µM, 1%), GGTI286 (10 µM, 1%), blebbistatin (10 µM, 0.02%), CT04 (2µg/mL, 1% glycerol) and Y27632 (10 µM, 0.1%). PC3 cells were incubated in inhibitor containing assay media for 30 minutes at 37° before adhesion measurements. One exception was made for CT04, which was used to incubate spreading PC3 cells 4 hours before adhesion measurements. Each dot represents the measurement of one PC3 cell attached to the cantilever via ConA (blue) or ephrin-A1-fc (brown). Indicated are contact times of PC3 cell and secondary substrate before being detached. The number of cells assayed for each condition is given by <n>. Bars mark mean force and standard deviation. For each contact time, the statistical differences to control experiments (untreated ephrin-A1 bound cells) were analyzed by Mann-Whitney *U*-tests (*P*-values given in gray).

a ephrin-A1 surface

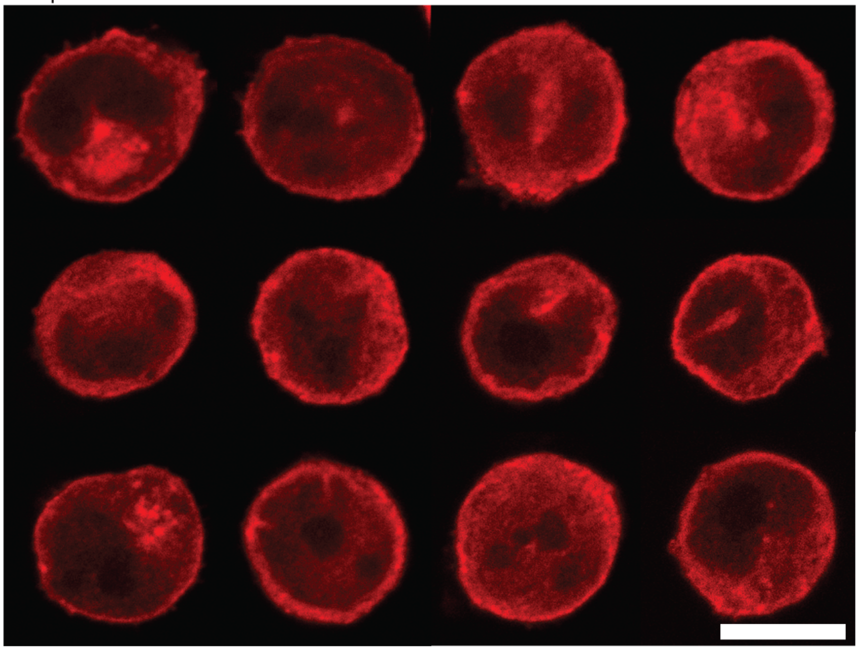

b ConA surface

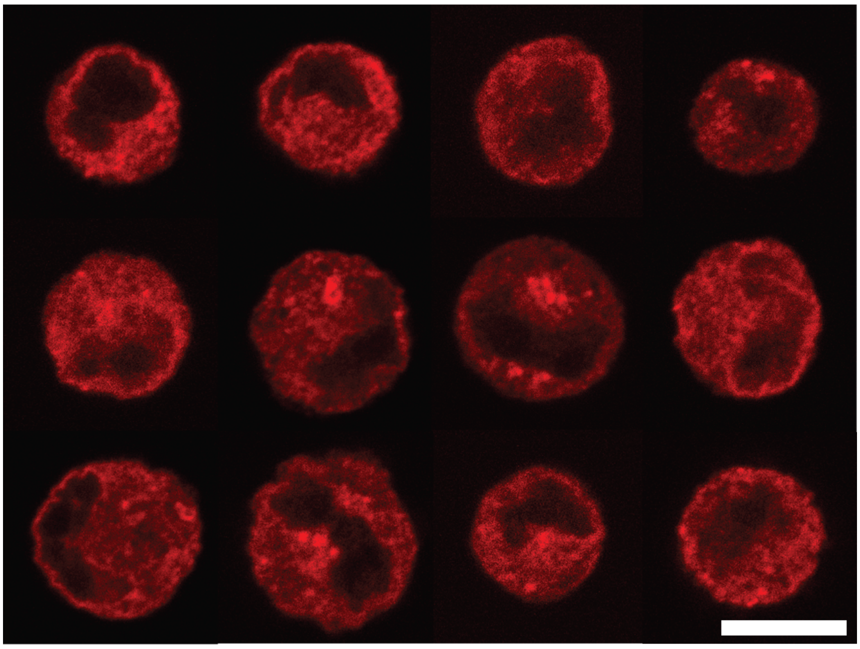

Supplementary Figure 8. Localization of β_1-integrin in PC3 cells bound to (a) ephrin-A1 and (b) ConA. PC3 cells were seeded for 1 h onto glass surfaces coat with (**a**) ephrin-A1-fc (50 µg/mL) or (**b**) ConA (2 mg/mL), fixed with 4% PFA for 20 minutes at 37°C, washed 3x with PBS, incubated in 0.1% Triton X-100 PBS for 20 minutes at room temperature, washed 3x with PBS, blocked with 2% BSA, 0.1% Triton X-100 in PBS (PBST) for 1 h at room temperature and incubated in PBST containing antibodies against integrin β_1-subunits (1:500) at 4°C overnight. Thereafter, the cells were washed 3x with PBS, incubated in AlexaFluor647-conjugated anti-rabbit IgG antibody (1:200, A21244 Life Technologies) PBST for 30 minutes at room temperature, washed 3x with PBS and finally covered with anti-fade (ProLong, Invitrogen). Confocal microscopy images were recorded with the focus plane in the middle of cell. Brightness and contrast were adjusted for all images. Scale bars, 10 µm.
